# Separable and Error-Free Reversible Data Hiding in Encrypted Image with High Payload

**DOI:** 10.1155/2014/604876

**Published:** 2014-04-06

**Authors:** Zhaoxia Yin, Bin Luo, Wien Hong

**Affiliations:** ^1^Key Laboratory of Intelligent Computing & Signal Processing, Ministry of Education, Anhui University, No. 111 Jiulong Road, Hefei 230601, China; ^2^Department of Information Management, Yu Da University, No. 168 Hsueh-fu Road, Tanwen Village, Zaoqiao Township, Miaoli County 36143, Taiwan

## Abstract

This paper proposes a separable reversible data-hiding scheme in encrypted image which offers high payload and error-free data extraction. The cover image is partitioned into nonoverlapping blocks and multigranularity encryption is applied to obtain the encrypted image. The data hider preprocesses the encrypted image and randomly selects two basic pixels in each block to estimate the block smoothness and indicate peak points. Additional data are embedded into blocks in the sorted order of block smoothness by using local histogram shifting under the guidance of the peak points. At the receiver side, image decryption and data extraction are separable and can be free to choose. Compared to previous approaches, the proposed method is simpler in calculation while offering better performance: larger payload, better embedding quality, and error-free data extraction, as well as image recovery.

## 1. Introduction

As a kind of technique of hiding data into cover media, for example, a digital image, data hiding might often produce a distorted version of the cover, known as stego-image. Based on whether the original cover can be reconstructed or not, data-hiding techniques can be classified into two kinds: irreversible [1, 2] and reversible [3–11]. Aiming at recovering the original image with no error, reversible data hiding is generally based on two technologies: difference expansion (DE) [3, 6, 7] and histogram shifting (HS) [5, 8–10]. In general, DE-based methods provide higher payload than HS-based methods at the sacrifice of image quality. As a result, HS-based methods are more popular when the image quality is an issue. Since the maximal embedding capacity of HS-based data-hiding methods equals the number of pixels in the peak point, more peak and zero pairs are required to enhance the payload. However, for most natural images, a few nonoverlapping peak and zero pairs could be found. Therefore, embedding capacity is limited.

Moreover, most of the existing reversible data-hiding methods are only suitable for unencrypted covers. However, in some application scenarios, content owners wish to encrypt the original images for maintaining secrecy or protecting privacy. Meanwhile, an inferior assistant or a channel administrator may desire to append some additional data within the cipher-text images without knowing the decryption key and the plaintext content. As a legal receiver, it is required that the original plaintext content can be recovered error-free after image decryption and data extraction. Reversible data hiding in encrypted image satisfies these needs [12–14].

In [13], data is appended by flipping three LSB of cipher-text image encrypted by simple exclusive-OR operation and extracted with the aid of spatial correlation in natural image. The original image can be recovered with no error when embedding payload is not too large. The performance is further improved by Hong et al. [14] using side match with a block-recovery order. In both [13, 14], the appended data can only be extracted after image decryption. In other words, a receiver having data-hiding key but no content-owner key cannot extract any information. To overcome this problem, a separable reversible data-hiding scheme [15] is proposed, in which the original image is encrypted using symmetric key and then data can be appended using data-hiding key. With an encrypted image containing additional data, a receiver having the data-hiding key can extract the appended data exactly, while a receiver having the symmetric key can decrypt the received data to obtain an image similar to the original one. If the receiver has both keys, he can extract the additional data and recover the original image at the same time.

Method [15] is undoubtedly a great idea. However, there is a limitation in the aspect of embedding payload. Data cannot be extracted exactly and the original image cannot be recovered completely when the payload is more than 0.04 bpp (this maximal value of effective payload may fluctuate along with different cover images). In other words, error appears and error rate rises as the payload increases. Beyond that, many parameters adopted in method [15] make it a little complicated for implementation. To solve these issues, we propose an improved work in this paper. After a cover image is being encrypted with a content-owner key, additional data can be embedded into the encrypted image based on block sorting and block histogram shifting with a data-hiding key. Compared with the existing methods, the proposed scheme has the following advantages: (1) simpler calculation and higher efficiency; (2) larger payload and better embedding quality; and (3) error-free recovery with high payload.

## 2. Proposed Scheme

The data extraction methods used in [13, 14] require estimating block smoothness. An incorrect estimation may result in the failure of data extraction and image recovery. Although a large block size reduces the extraction error rate, it decreases the payload as well.

The proposed method embeds data by shifting pixels locally in encrypted image. The content owner partitions the cover image into nonoverlapping blocks and encrypts the cover image using multigranularity encryption: coarse-grained encryption permutes blocks in global images while fine-grained encryption permutes the pixels in each block to construct a meaningless encrypted image. Although all pixels are permuted, pixels in each block still preserve the same image histogram. Therefore, the HS method is applicable for embedding data into permuted blocks if pairs of peaks and zeros in each block are properly determined. In our approach, two basic pixels are randomly selected from the permuted block and used to indicate two peak points. Since the pixels having the same values as peaks contribute to the payload, it is likely to embed more than one bit per block to achieve high payload. More importantly, the embedded data bits can be extracted exactly. In addition, since the values of basic pixels are preserved during data embedding, they can be exploited to estimate the smoothness of blocks roughly and indicate the priority of embedding sequence.

**Figure 1:**
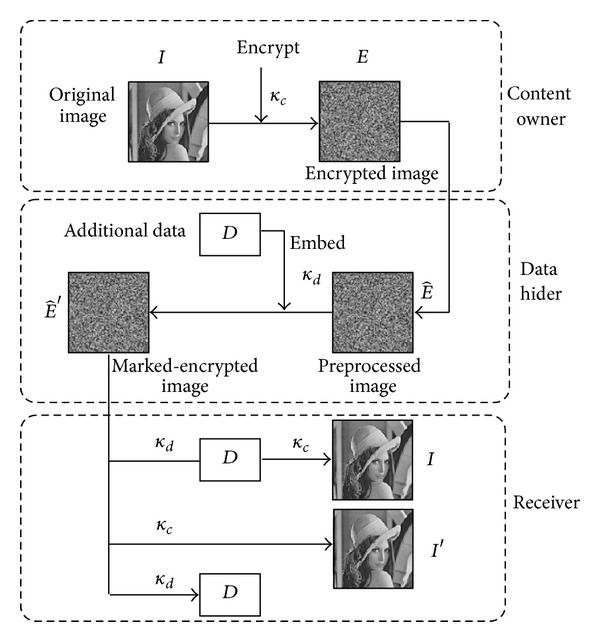
Framework of the proposed method.

The proposed method is described briefly as follows. In image encryption and data embedding phase, the content owner encrypts the original image *I* using a symmetric content-owner key *κ*_*c*_ to produce an encrypted image *E*. Then, the data-hider processes image *E* to generate image $\hat{E}$. Additional data *D* is embedded into $\hat{E}$ with data-hiding key *κ*_*d*_ and marked-encrypted image ${\hat{E}}^{\prime }$ is obtained. In data extraction and image recovery phase, there are three options for legal receivers. Image decryption and data extraction are separable and can be free to choose. The embedded data *D* can be easily extracted from ${\hat{E}}^{\prime }$ with *κ*_*d*_. Since only part of pixels is modified by one grayscale unit to conceal *D*, direct decryption on ${\hat{E}}^{\prime }$ with *κ*_*c*_ generates a decrypted image *I*′, which is very similar to the original version *I*. If *κ*_*c*_ and *κ*_*d*_ are both adopted, the cover image *I* can be restored error-free and the embedded data *D* can be extracted accurately. The framework of the proposed method is shown in Figure 1.

The goal of the proposed method is to improve embedding payload, quality, and efficiency via simple calculation. The last and most important, keep error-free recovery as the payload increases while [13–15] cannot.

### 2.1. Image Encryption

Firstly, the cover image *I* is divided into *N* nonoverlapping blocks {*B*_*i*_}_*i*=0_^*N*−1^. Each block *B*_*i*_ is composed of *m* × *n* pixels. Then, multigranularity encryption is adopted by using random permutation to obtain the permuted blocks ${\left\{{\hat{B}}_{i}\right\}}_{i=0}^{N-1}$: pixels permutation in each block and blocks permutation in the whole cover image. Thus, the encrypted image *E* is generated. Parameters *m* and *n* and the integer *s*_*c*_ adopted as the seed of random permutation compose the content-owner key *κ*_*c*_.

### 2.2. Data Embedding

After receiving *E* together with block size *m* × *n*, the data hider partitions *E* into *N* blocks ${\left\{{\hat{B}}_{i}\right\}}_{i=0}^{N-1}$. For each block ${\hat{B}}_{i}$, two basic pixels ${\hat{b}}_{i,L}$ and ${\hat{b}}_{i,R}$ are randomly selected and other *m* × *n* − 2 pixels are denoted by  ${\left\{{\hat{q}}_{i,j}\right\}}_{j=0}^{m\times n-3}$; that is, ${\hat{B}}_{i}={\left\{{\hat{b}}_{i,L},{\hat{b}}_{i,R},{\hat{q}}_{i,j}\right\}}_{j=0}^{m\times n-3}$. Next, to estimate the smoothness of each block, the difference ${\hat{d}}_{i}=|{\hat{b}}_{i,R}-{\hat{b}}_{i,L}|$ is calculated. Blocks with smaller ${\hat{d}}_{i}$ are likely smoother than blocks with larger ${\hat{d}}_{i}$, and it is known that smoother blocks are in favor of HS. As a result, blocks with smaller ${\hat{d}}_{i}$ will be chosen to have higher priority for carrying data. Let ${\left\{{\hat{d}}_{\varphi (i)}\right\}}_{i=0}^{N-1}$ be the sorted result of ${\left\{{\hat{d}}_{i}\right\}}_{i=0}^{N-1}$ after being sorted in the ascending order. The sorted sequence {*φ*(*i*)}_*i*=0_^*N*−1^ is then employed as the embedding sequence of blocks. At last, two peaks ${\hat{P}}_{i,L}$ and ${\hat{P}}_{i,R}$ in each block are determined as follows: ${\hat{P}}_{i,L}=\min({\hat{b}}_{i,L},{\hat{b}}_{i,R})$,  ${\hat{P}}_{i,R}=\max({\hat{b}}_{i,L},{\hat{b}}_{i,R})$. To ensure that each block has two distinct peaks, we simply set ${\hat{p}}_{i,R}={\hat{p}}_{i,L}+1$ when ${\hat{p}}_{i,L}={\hat{p}}_{i,R}$.

To avoid saturated pixels (pixels valued 0 or 255) from overflow or underflow during embedding, saturated pixels have to be preprocessed by modifying one grayscale unit and noted in a location map *L*. To do this, visit blocks ${\left\{{\hat{B}}_{\varphi (i)}\right\}}_{i=0}^{N-1}={\left\{{\left\{{\hat{b}}_{\varphi (i),L},{\hat{b}}_{\varphi (i),R},{\hat{q}}_{\varphi (i),j}\right\}}_{j=0}^{m\times n-3}\right\}}_{i=0}^{N-1}$ sequentially and append a bit “1” to *L* when ${\hat{q}}_{\varphi (i),j}\in \{1,254\}$. If ${\hat{q}}_{\varphi (i),j}\in \{0,255\}$, append a bit “0” to *L* and modify ${\hat{q}}_{\varphi (i),j}$ to ${\hat{q}}_{\varphi (i),j}^{\prime }$ using the following equation:

(1) $$\begin{array}{c} q_{\varphi (i),j}^{\prime }=\{\begin{array}{cc} 254, & q_{\varphi (i),j}=255\\ 1, & q_{\varphi (i),j}=0\\ q_{\varphi (i),j}, & \text{otherwise}. \end{array} \end{array}$$

Let the processed block be *B*_*φ*(*i*)_′. The embedding capacity of *B*_*φ*(*i*)_′, denoted by *C*_*i*_ (bits), equals the number of nonbasic pixels valued ${\hat{p}}_{\varphi (i),L}$ and ${\hat{p}}_{\varphi (i),R}$. Continue the preprocessing procedures until the condition ∑_*i*=0_^*M*−1^ *C*_*i*_ ≥ |*L* | +|*D*| is satisfied, where *D* is the additional data and *M* is the minimal number of blocks that are used for embedding *L* and *D*. We denote the preprocessed encrypted image by  $\hat{E}$. Once $\hat{E}$ is obtained, the data hider concatenates *L* and *D* to form a string of message bits *S* and then scans the pixels {{*q*_*φ*(*i*),*j*_′}_*j*=0_^*m*×*n*−3^}_*i*=0_^*M*−1^ in {*B*_*φ*(*i*)_′}_*i*=0_^*M*−1^ to conceal *S* as follows. If the scanned pixel *q*_*φ*(*i*),*j*_′ is valued ${\hat{p}}_{\varphi (i),L}$ or ${\hat{p}}_{\varphi (i),R}$, a bit *s* ∈ {0,1} extracted from *S* is embedded by modifying *q*_*φ*(*i*),*j*_′ to *q*_*φ*(*i*),*j*_′′ according to the following equation:

(2) $$\begin{array}{c} q_{\varphi (i),j}^{\prime \prime }=\{\begin{array}{cc} q_{\varphi (i),j}^{\prime }-s, & q_{\varphi (i),j}^{\prime }=p_{\varphi (i),L}\\ q_{\varphi (i),j}^{\prime }+s, & q_{\varphi (i),j}^{\prime }=p_{\varphi (i),R}. \end{array} \end{array}$$

Otherwise, pixels are either maintained or shifted by one unit using the following equation:

(3) $$\begin{array}{c} q_{\varphi (i),j}^{\prime \prime }=\{\begin{array}{cc} q_{\varphi (i),j}^{\prime }, & p_{\varphi (i),L}<q_{\varphi (i),j}^{\prime }<p_{\varphi (i),R}\\ q_{\varphi (i),j}^{\prime }-1, & q_{\varphi (i),j}^{\prime }<p_{\varphi (i),L}\\ q_{\varphi (i),j}^{\prime }+1, & q_{\varphi (i),j}^{\prime }>p_{\varphi (i),R}. \end{array} \end{array}$$

After embedding, blocks {*B*_*φ*(*i*)_′}_*i*=0_^*M*−1^ are modified to {*B*_*φ*(*i*)_′′}_*i*=0_^*M*−1^, and the marked-encrypted image ${\hat{E}}^{\prime }$ is generated. The parameters *m*,  *n*,  |*L* | ,  |*S*| and the seed *S*_*d*_ used to randomly select basic pixels compose the data-hiding key *κ*_*d*_.

### 2.3. Data Extraction and Image Recovery

If the receiver has data-hiding key *κ*_*d*_, the embedded additional data *D* can be extracted directly from the marked-encrypted image ${\hat{E}}^{\prime }$. To extract *D*, ${\hat{E}}^{\prime }$ is firstly partitioned into blocks ${\left\{B_{i}^{\prime \prime }\right\}}_{i=0}^{N-1}={\left\{{\left\{{\hat{b}}_{i,L},{\hat{b}}_{i,R},{\hat{q}}_{i,j}^{\prime \prime }\right\}}_{j=0}^{m\times n-3}\right\}}_{i=0}^{N-1}$ sized *m* × *n*. Peaks ${\hat{p}}_{\varphi (i),L}$ and ${\hat{p}}_{\varphi (i),R}$ and the differences ${\left\{{\hat{d}}_{i}\right\}}_{i=0}^{N-1}={\left\{|{\hat{b}}_{i,R}-{\hat{b}}_{i,L}|\right\}}_{i=0}^{N-1}$ are then determined. Block smoothness ${\left\{{\hat{d}}_{i}\right\}}_{i=0}^{N-1}$ is sorted in ascending order and the result is denoted by ${\left\{{\hat{d}}_{\varphi (i)}\right\}}_{i=0}^{N-1}$. The sorted sequence {*φ*(*i*)}_*i*=0_^*N*−1^ is then employed as the extracting sequence of blocks. At last, the embedded data can be extracted from pixels ${\left\{{\hat{q}}_{\varphi (i),j}^{\prime \prime }\right\}}_{j=0}^{m\times n-3}$ in block *B*_*φ*(*i*)_′′ using the following equation:

(4) $$\begin{array}{c} s=\{\begin{array}{cc} 0, & {\hat{q}}_{\varphi (i),j}^{\prime \prime }={\hat{p}}_{\varphi (i),L}\;\;\text{or}\;\;{\hat{q}}_{\varphi (i),j}^{\prime \prime }={\hat{p}}_{\varphi (i),R}\\ 1, & {\hat{q}}_{\varphi (i),j}^{\prime \prime }={\hat{p}}_{\varphi (i),L}-1\;\;\text{or}\;\;{\hat{q}}_{\varphi \left(i\right),j}^{\prime \prime }={\hat{p}}_{\varphi (i),R}+1. \end{array} \end{array}$$

The first |*L*| extracted bits compose the location map *L*, and the other |*D*| bits compose the additional data *D*. If the receiver also has *κ*_*c*_, original cover image *I* can be perfectly recovered by firstly restoring the pixels  ${\hat{q}}_{\varphi (i),j}^{\prime }$ from ${\hat{q}}_{\varphi (i),j}^{\prime \prime }$ using the following equation:

(5) $$\begin{array}{c} {\hat{q}}_{\varphi (i),j}^{\prime }=\{\begin{array}{cc} {\hat{q}}_{\varphi (i),j}^{\prime \prime }, & {\hat{p}}_{\varphi (i),L}<{\hat{q}}_{\varphi (i),j}^{\prime \prime }<{\hat{p}}_{\varphi (i),R}\\ {\hat{q}}_{\varphi (i),j}^{\prime \prime }+1, & {\hat{q}}_{\varphi (i),j}^{\prime \prime }<{\hat{p}}_{\varphi (i),L}\\ {\hat{q}}_{\varphi (i),j}^{\prime \prime }-1, & {\hat{q}}_{\varphi (i),j}^{\prime \prime }>{\hat{p}}_{\varphi (i),R}. \end{array} \end{array}$$

To recover ${\left\{{\hat{q}}_{\varphi (i),j}\right\}}_{i=0}^{M-1}$ from ${\left\{{\hat{q}}_{\varphi (i),j}^{\prime }\right\}}_{i=0}^{M-1}$, if ${\hat{q}}_{\varphi (i),j}^{\prime }\in \{1,254\}$, extract a bit *b* from *L*. If the extracted bit *b* = 1, set ${\hat{q}}_{\varphi (i),j}={\hat{q}}_{\varphi (i),j}^{\prime }$. Otherwise; that is, *b* = 0, set ${\hat{q}}_{\varphi (i),j}=0$ when ${\hat{q}}_{\varphi (i),j}^{\prime }=1$; set ${\hat{q}}_{\varphi (i),j}=255$ when ${\hat{q}}_{\varphi (i),j}^{\prime }=254$. Repeat until the encrypted image $E={\left\{{\hat{B}}_{\varphi (i)}\right\}}_{i=0}^{N-1}={\left\{{\hat{B}}_{i}\right\}}_{i=0}^{N-1}={\left\{{\left\{{\hat{p}}_{i,L},{\hat{p}}_{i,R},{\hat{q}}_{i,j}\right\}}_{j=0}^{m\times n-3}\right\}}_{i=0}^{N-1}$ is reconstructed accordingly. With the content owner key *κ*_*c*_, *E* can be exactly decrypted to the original cover image *I*. Note that if the receiver only has *κ*_*c*_ but no *κ*_*d*_, an image *I*′ that is very similar to the original one can be obtained.

## 3. Experimental Results

**Figure 2:**
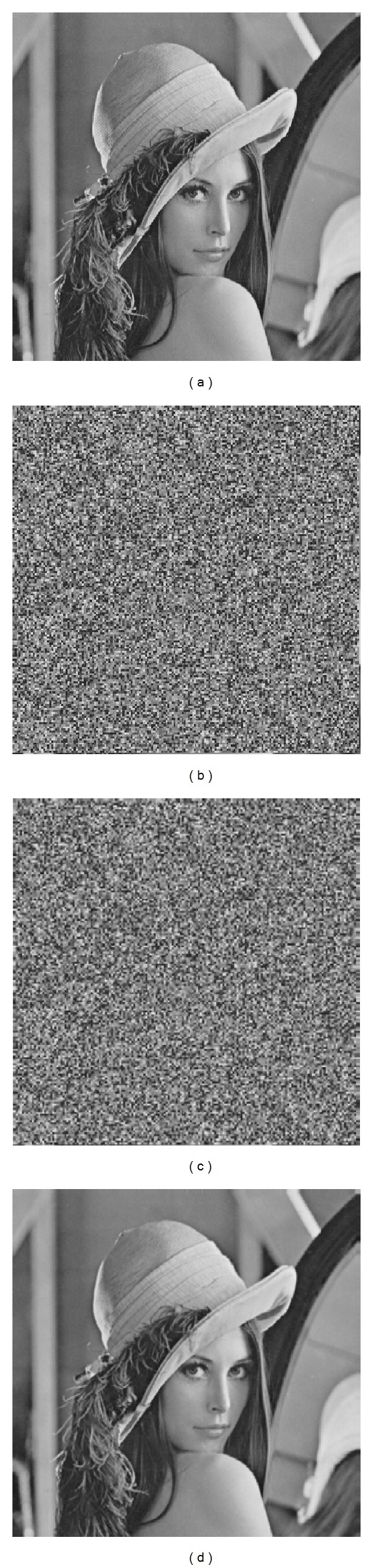
(a) Original Lena and error-free recovered version, (b) encrypted version, (c) stego-encrypted image containing additional data with embedding rate 0.13 bpp, and (d) directly decrypted version with PSNR 50.51 dB.

A number of gray images sized 512 × 512 were used as original cover images in our experiment. Figures 2(a) and 2(b) show the original image Lena and its encrypted version (*m* = 4, *n* = 4). After embedding 33910 bits of additional data into Figure 2(b), the stego-encrypted image was obtained, as shown in Figure 2(c) in which the embedding rateis 0.13 bpp. With the image shown as Figure 2(c), the receiver having the data-hiding key could extract the embedded data from it. The directly decrypted image only using the symmetric cryptographic key is given as Figure 2(d), and the value of PSNR between (a) and (d) is 50.51 dB. Using both the data-hiding key and cryptographic key, we successfully extracted the additional data and recovered the original image error-free.

Table 1 summarizes the embedding payloads, PSNR in directly decrypted images (PSNRdec), and PSNR in recovered images (PSNRrec) when different block sizes were used for image Lena. Each “+*∞*” in Table 1 indicates that the mean square errors between the recovered version and the original image are 0 and the cover was reconstructed error-free.

**Figure 3:**
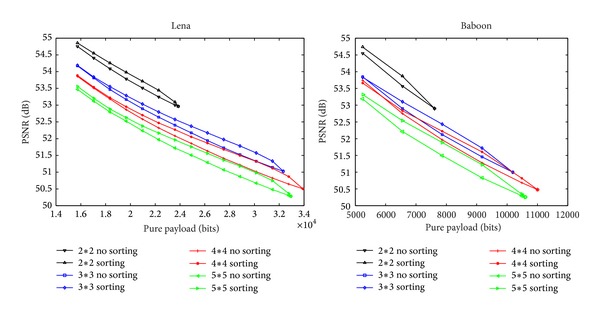
Performance of different strategies with different parameters.

To explore the influence of different parameters (block sizes *m* × *n*) and different strategies (block sorting or no sorting) on embedding performance of proposed method, we compared different results obtained from a smooth image Lena and a complex image Baboon. In order to obtain enough experimental data and assure validity of conclusions, for each test image, 4 block sizes (2 × 2,3 × 3,4 × 4,5 × 5) are adopted. For each block size, 10 integers are chosen as *s*_*c*_ to generate different *E*. Then, embed 10 distinct *D* into each *E* and cross-test 100 times. After removal of the highest and lowest points, take the average to investigate PSNR-payload curves. Take Lena and Baboon as examples shown in Figure 3. The abscissa represents the pure embedding payload and the ordinate is the value of PSNR between *I* and *I*′.

From Figure 3 we can draw some conclusions. (1) The smaller the block size, the better the PSNR. If embedding quality is preferable, block size 2 × 2 is good choice. (2) When block size is larger than 4 × 4, the performance would be worse. If large payload is desirable, either 4 × 4 or 3 × 3 could be chosen. (3) Under the same block size, the performance of block sorting strategy is better than that of no sorting strategy. Taking image Lena as an example, with 26214 bits of pure payload and 3 × 3 block size, the PSNR is 52.17 dB (sorting) and 51.94 dB (no sorting), respectively. Looking into the reason, smoother blocks are in favor of HS and have higher priority for carrying data in block sorting strategy. So, for appending the same amount of bits into the same encrypted image based on HS, fewer pixels are modified by using block sorting strategy. That is the reason leading to higher PSNR. (4) Smooth image Lena provides better performance than complex one, Baboon. It is known that, for HS-based methods, smoother blocks often provide larger capacity than complex ones. Therefore, the full payload of Lena is larger than that of Baboon. For the same nonfull payload, fewer blocks are used in Lena and embedding distortion is smaller. So PSNR is higher.

**Figure 4:**
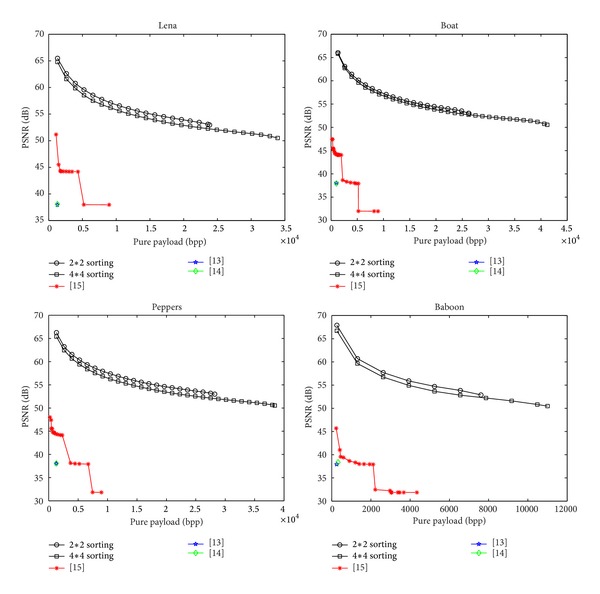
Rate-distortion performance between different approaches.

We compared the proposed scheme with methods [13–15] in Figure 4, which indicates that the proposed scheme has the best performance. All results are derived from the best parameters under a condition that the original image can be recovered without any error.

Note that the data extraction in [13, 14] is not separable from the content decryption. However, using the proposed scheme or method [15], data extraction and image encryption are separable and can be free to choose. With the proposed scheme, since both the similarity of neighboring pixels in local level and block smoothness are fully exploited, more redundant space can be created to carry data. So the performance curve of the proposed scheme is better than those of other methods.

We also compared the proposed scheme under the same block size with the nonseparable method in [13, 14]. The results are shown in Table 2, from which we see that the proposed scheme has 2 times gain of payload under the block size 4 × 4 and 7 times gain under block size 8 × 8 together with improvement of PSNR value in directly decrypted image when meeting the perfect recovery condition.

**Figure 5:**
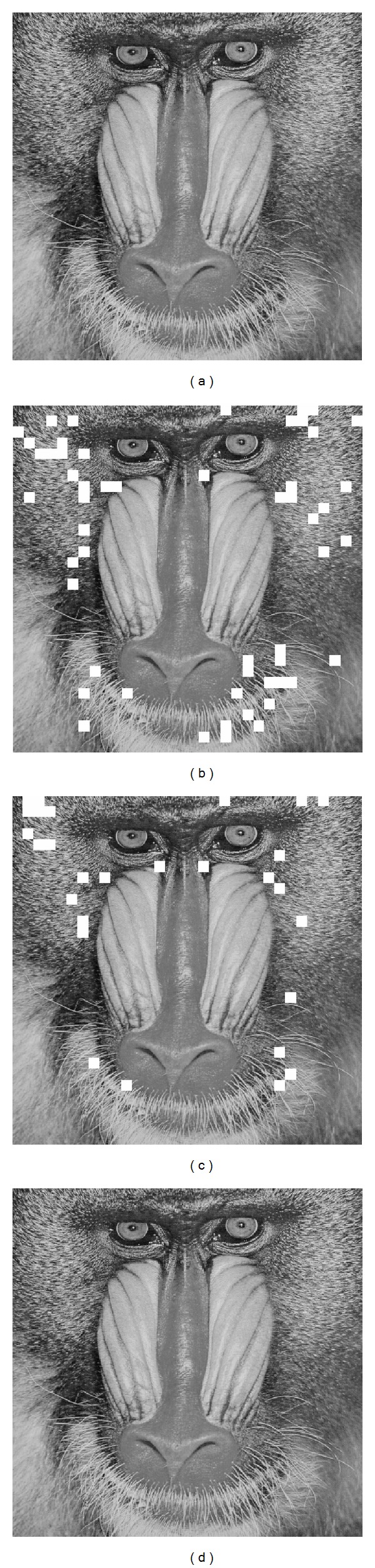
(a) Original Baboon, (b) recovered version by [13], (c) recovered version by [14], and (d) recovered version by proposed method.

Furthermore, we take Baboon as an example to verify details. Under the same block size 8 × 8, we compare recovered images generated by different methods in Figure 5, where the incorrect recovered blocks are marked by white.

Comparing Figures 5(b), 5(c), and 5(d), we see that the proposed method recovers the image blocks error-free and more accurate than that of [13, 14]. Although the experiments were based on Baboon, experiments on other test images also showed the similar result, which indicates that the proposed method offers a better performance for data extraction and image recovery.

Finally, we summarize maximum payload and corresponding PSNR of Lena, Peppers, Boat, and Baboon in Table 3. For the same cover image, the maximum payload of the proposed scheme is much more than that of [13–15] and the embedding quality is the best.

## 4. Conclusion

This paper proposed a separable and error-free reversible data-hiding scheme in encrypted image, which significantly outperforms the previous methods in the three aspects of payload, PSNR, and error rate. Compared with [13, 14], not only can cover images be reconstructed with no error, but also image decryption and data extraction are separable. Compared with [15], the proposed method improves both PSNR and the effective payload via simpler calculation using few parameters and achieves higher efficiency. The last and most important advantage of our method is that it can keep error-free recovery as the payload increases while the others cannot.

## Figures and Tables

**Table 1:** Maximum payload, PSNR in directly decrypted images (PSNRdec) and PSNR in recovered images (PSNRrec) when different block size was used for image Lena.

Block Size	Maximum Payload		Image Quality	
	bpp	Bits	PSNRdec	PSNRrec
2 × 2	0.091	23855	52.96	+*∞*
3 × 3	0.123	32328	51.03	+*∞*
4 × 4	0.129	33910	50.51	+*∞*
5 × 5	0.126	32943	50.28	+*∞*
8 × 8	0.114	29870	50.07	+*∞*

**Table 2:** Comparison of performance in the three aspects of Payload, PSNR and Error rate (ER) between different approaches on Lena.

	Block Size							
	4 × 4				8 × 8			
	Payload (bits)	PSNRdec (dB)	PSNRrec (dB)	Error rate (%)	Payload (bits)	PSNRdec (dB)	PSNRrec (dB)	Error rate (%)
[13]	16384	37.93	43.49	15.26	4096	37.93	54.80	1.10
[14]	16384	37.94	51.91	4.74	4096	37.93	59.02	0.42
Proposed	33910	50.51	+*∞*	0	29870	50.07	+*∞*	0

**Table 3:** Maximum payload and corresponding PSNR values.

Method	Lena		Peppers		Boat		Baboon	
	payload	PSNR	payload	PSNR	payload	PSNR	payload	PSNR
[13]	1024	37.94	1296	38.03	1024	38.06	256	37.92
[14]	1296	38.08	1296	38.05	1024	37.93	324	38.37
[15]	8956	37.96	8956	31.82	8956	31.96	4340	31.86
Proposed	33910	50.51	38420	50.54	41194	50.54	11004	50.48
